# Should a single growth standard be used to judge the nutritional status of children under age 5 years globally: Yes

**DOI:** 10.1016/j.ajcnut.2024.04.019

**Published:** 2024-09-10

**Authors:** Elaine Borghi, Harshpal Singh Sachdev

**Affiliations:** 1 Department of Nutrition and Food Safety, World Health Organization, Geneva, Switzerland; 2 Department of Pediatrics and Clinical Epidemiology, Sitaram Bhartia Institute of Science and Research, New Delhi, India

**Keywords:** child growth, global standards, growth standards, child health, equity

## Abstract

Childhood nutritional status serves as a lens through which nations and communities identify missed opportunities to improve health and wellbeing across the life cycle, as well as economic development and other related sectors. Countries have committed to the global nutrition targets endorsed by the World Health Assembly in 2012, which were included in the Sustainable Development Goals framework under the target to end all forms of malnutrition by 2030. The child malnutrition indicators for tracking countries’ progress toward the agreed-upon targets are based on standard definitions of nutritional status against the widely adopted and used World Health Organization (WHO) Child Growth Standards. The standards were based on a sample of healthy breastfed infants and young children from diverse ethnic backgrounds and cultural settings as part of the WHO Multicentre Growth Reference Study. The WHO Child Growth Standards developed represent the best description of physiological growth for children aged <5 y. The standards depict normal early childhood growth under optimal environmental conditions and can be used to assess children everywhere, regardless of ethnicity, socioeconomic status, and type of feeding.

## Main Argument (Borghi)

Childhood nutritional status serves as a lens through which nations and communities identify missed opportunities to improve health and wellbeing across the life cycle as well as economic development and other related sectors. Although it is well known that the greatest benefits result from improving nutrition in the early stages of life, the so-called “window of opportunity” [1], a life-course perspective to improving nutrition is also needed, targeting older children and adolescents beyond infants and young children. Countries should be provided appropriate tools to assess their children’s nutritional status to identify and tackle the causes of malnutrition, thus improving health and wellbeing across the entire life cycle. Having such tools based on optimal growth patterns depicted by children living under nearly ideal environmental conditions and given appropriate feeding is essential to ensure that all children, regardless of their place of birth, achieve their full growth potential. The WHO Multicentre Growth Reference Study (MGRS) proved this is possible when children from 6 different sites showed they grow linearly strikingly similarly [2].

In 2012, the World Health Assembly endorsed the comprehensive implementation plan on maternal, infant, and young child nutrition to guide and encourage countries toward improving their population’s nutritional status [3]. The plan included 6 targets to be achieved by 2025, 3 of them related to child malnutrition, namely stunting, wasting, and overweight. The Sustainable Development Goals (SDG) Target 2.2 to eliminate all forms of malnutrition incorporated the 2025 global nutrition targets and included in its accountability framework the 3 child malnutrition targets. The SDG agenda was accepted by all countries and is applicable to all, considering different national realities, capacities, and levels of development and respecting national policies and priorities [4].

The child malnutrition indicators for tracking countries’ progress toward the agreed-upon targets are based on standard definitions of nutritional status against the widely adopted and used WHO Child Growth Standards [5].

### WHO Child Growth Standards

In 1995, WHO convened the Working Group on Infant Growth as part of a larger effort initiated by WHO to evaluate the interpretation and appropriate uses of anthropometric data in all age groups. Based on a review of the previously recommended National Center for Health Statistics/WHO international growth references, the group concluded that, in addition to technical limitations of the data and methodology used, the growth reference did not describe infants’ physiologic growth adequately; importantly, breastfed children patterns were misrepresented by those references, undermining breastfeeding functional benefits. The group advised that new references be developed based on a prescriptive approach, which would constitute standards to describe the growth pattern of healthy breastfed infants. The new standards were thus to describe how children should grow to achieve their full growth potential in all countries rather than merely describe how they actually grew at a given time and place [6,7].

The WHO MGRS collected growth data and related information from 8440 affluent children from widely differing ethnic backgrounds and cultural settings (Brazil, Ghana, India, Norway, Oman, and the United States). Eligibility criteria included breastfeeding, no maternal smoking, and environments supportive of unconstrained growth [2]. The new curves provide a better description of physiologic growth and reveal that intrauterine retardation in length is a greater problem than previously believed, and thus the need for greater efforts to improve nutritional status of pregnant women and women of childbearing age [1]. The introduction of the WHO Child Growth Standards catalyzed other important advances in growth monitoring, highlighting the importance of a worldwide tool to identify growth faltering and help countries reduce malnutrition and attend to severe crises. In addition to promoting equity across countries, with one reference measure to all children in the world targeting optimal individual growth, there are important features that allow for early and efficient ways to detect growth faltering. The switch from using only weight-for-age to using multiple indices to better characterize growth patterns, especially the monitoring of length and height, is crucial, because faltering patterns are clearly different for weight-for-age and length- or height-for-age and are associated with different outcomes. This advance was particularly important to avoid imbalances between growth in weight and in length or height. Moreover, the introduction of the index weight-for-length or height significantly enhanced the assessment of severe acute malnutrition [8,9] (i.e., wasting) as well as overweight and obesity [8].

### The use of a single standard globally

The use of a single, global growth standard to assess nutrition status of children from different countries and ethnicities is a powerful and effective tool for underscoring the right for every child to grow to its full potential when their nurturing follows health recommendations and care practices associated with healthy outcomes [10]. Compared with the previously recommended National Center for Health Statistics/WHO international growth reference, the new WHO standards provide a tool to monitor the rapid and changing rate of growth in early infancy, which is based on a more accurate description of optimal growth. Their adoption and use have important implications for child health with respect to the assessment of lactation performance and the adequacy of infant feeding [11]. The MGRS showed striking similarity in linear growth among children in the 6 sites, which justifies pooling the data and constructing a single international standard from birth to 5 y of age. Variance components analyses demonstrated that variability in growth was due overwhelmingly to differences among individuals (70% of the total variance) and only minimally to differences among sites (3% of the total variance). The choice of length/height for assessing the differences of possible genetic or environmental origin among children of well-off families was based on the following arguments. Linear growth is normally distributed and resistant to skewness in response to excessive energy intakes, unlike weight which is more plastic in response to overnutrition. Although linear growth can be affected negatively and profoundly by environmental factors such as diet and infection, these were not relevant in the affluent populations selected for the MGRS study [12]. The indicator of poor linear growth, stunting, was shown to be a useful metric for comparing the environmental deficits to which children are exposed between populations and over time [13,14].

Ensuring the best possible environment for mothers before conception to reduce the incidence of low-birth weight will help to break the intergenerational cycle of malnutrition. Management of childhood overweight also requires action throughout the school years [15]. The 2013 Lancet Maternal and Child Nutrition Series highlighted various interventions across the life course that are expected to have the largest impact on tackling undernutrition and overweight. Regardless of secular trends, several studies point to normal growth can be achieved in children born to mothers who were malnourished in childhood when profound improvements in health, nutrition, and the environment take place before conception [16]. The MGRS confirmed that similar growth can be achieved in settings with widely disparate parental heights if health recommendations and care practices associated with healthy outcomes were ensured [2,17]. This observation and favorable comparisons between midparental heights and the children’s projected adult height (based on children’s height at 2 y of age) support that the prescribed care that characterized the MGRS populations (e.g., exclusive or predominant breastfeeding, implementation of recommended complementary feeding practices, and adoption of recommended pediatric care) eliminated or minimized significant environmental causes that account for substantial population-level differences in child growth seen globally. The favorable socioeconomic status of families included in the study is also likely to have resulted in adequate nutritional status of mothers before and during pregnancy. These results show that, notwithstanding adverse conditions likely experienced by their parents, within one generation, communities can attain significant advances in children’s linear growth, ≥2 y of age, when care and nutrition approximate international recommendation [17], which can be justifiably extended to ≤5 y of age.

The intergenerational cycle of growth failure must be broken by improving socioeconomic, nutrition, and health conditions. Sustained provision of nutrition and environmental conditions that offer unconstrained growth, allowing children to achieve their full growth potential, can break the intergeneration cycle of malnutrition. The use of one global standard to judge the nutrition status of children aged <5 y across the globe will not only ensure each child the right to equitable growth but will also ensure the rights of its future generations.

In conclusion, 17 y after their release, the WHO standards have been thoroughly scrutinized and widely implemented. According to a survey carried out in 2011, 125 countries had officially adopted the WHO standards. Since then, WHO has consistently supported countries to implement them and interpret their uses. This is consistent with WHO documentation received from countries marked as “under revision” back in 2011, that came forward indicating adoption later, adding to above 160 countries since our last count. The time between adoption and implementation varies among countries, depending on resources available to follow WHO recommendations for the uses of the standards. Nevertheless, at global level, child malnutrition SDG data custodians, namely WHO and UNICEF, use the standards to monitor countries’ progress toward the targets related to childhood nutrition. The WHO Child Growth Standards represent the best description of physiological growth and should be applied to all children everywhere, regardless of ethnicity, socioeconomic status, and type of feeding.

## Refutation (Sachdev)

Evidence directly relevant to the topic of the debate is conspicuous by its absence; namely, proof of the utility of a single growth standard, in children aged <5 y, to consistently predict a biological construct representing the balance between nutritional intake and requirement or precisely identify the well characterized phenotype of adequate or inadequate nutritional status. Childhood growth is influenced by several factors, among which nutrition is only one, and its contribution to child growth can vary across contexts and time. As an illustration, despite comparable body weights, healthy Indian infants have proportionately lower lean tissue and greater fat mass compared with other ethnicities [18], and in a recent Indian survey [19] that used the current WHO standards, half of the anthropometrically undernourished 5–19-y-old children (thin or BMI for age below −2*Z* 54%, stunted or height-for-age below −2*Z* 59%) showed “metabolic obesity” (dysglycemia or dyslipidemia). Thus, if the diagnostic test accuracy of a single growth standard to predict phenotypically well characterized malnutrition using precise biomarkers were uniform in different contexts, it would have provided clinching evidence in favor of the debate motion. Unfortunately, that is not so.

The Main Argument that has been provided revolves around proving that globally, growth in children aged <5 y is “strikingly similar” under optimal environmental conditions. In other words, body size is being directly equated with the biological construct of nutritional status. Further, using linear growth as the sole dimension to determine similarity of childhood growth is suspect. This decision is justified with the following quote: “linear growth is normally distributed and resistant to skewness in response to excessive energy intakes, unlike weight which is more plastic in response to overnutrition. Although linear growth can be affected negatively and profoundly by environmental factors such as diet and infection, these were not relevant in the affluent populations selected for the MGRS study.” However, the selected affluent populations may be more susceptible to overnutrition, which should have been an equal, if not a greater concern, for determining the optimal nutritional status in the current era. Thus, demonstration of homogeneity of weight-related indices (weight-for-age and weight-for-stature) and other dimensions such as head circumference is imperative for concluding “striking similarity” in growth. In fact, systematic reviews show that variability in weight-for-age and head circumference is greater than linear growth [[20], [21], [22]]. Unfortunately, such evidence is not presented or available for scrutiny in the public domain. Finally, the quantification criteria [2] for concluding “striking similarity” of linear growth, namely, within ±0.5 SD differences between the mean and individual sites, are too lenient, as detailed in my Main Argument. With ∼27% unmeasured variance, a substantially greater interindividual variability (70%) compared with that between the limited 6 sites (3%) is not surprising but still compatible with practically relevant differences (≥0.2−0.5 SD) between any 2 sites.

Because the “…best possible environment for mothers before conception...” is invoked as a unifying concept for the global standard, it is reasonable to consider the site-based heterogeneity of fetal growth curves as well. Fetal growth is measured as the estimated fetal weight (EFW). Compared with ecologic site variations in the postnatal growth environment, the intrauterine *milieu interieur* is tightly and homeostatically regulated; therefore, intersite variance in fetal growth should be negligible and certainly lower than the intersite variance that has been recorded in postnatal growth [2]. However, it has recently been argued that there is no “universal reference” for fetal growth since, despite similar inclusion criteria, EFW percentiles for gestational age vary among studies, which result in different proportions of fetuses as being classified below or above a cutoff point [23]. The WHO Fetal Growth study was conducted in women with no environmental or economic constraints likely to impede fetal growth [24]. An examination of the site growth curves revealed significant site (country) differences at all percentiles and gestational ages, which were not only dependent on maternal characteristics and fetal sex but also independent of these variables. The difference in EFW between some country sites was >50 g at the 10th percentile at the 28th week of gestation, which might be clinically relevant, and pooling data could result in the misdiagnosis of both small- and large-for-gestational-age babies. Notably, there were differences in birthweight between countries; for example, India had significantly smaller neonates than the other countries, even after adjusting for gestational age. The median birthweights in the 10 sites ranged between 2975 g and 3575 g —a difference of 600 g! Thus, the expectation of uniform underfive growth as a standard among healthy babies in various countries is problematic.

Adequate consideration has not been given to the external validation of the MGRS findings and the potential adverse consequences of the practical application of a global and uniform growth standard, especially in judging the nutritional status of children aged <5 y (summarized in greater detail in my Main Argument). The advantages of simplicity or setting an equitable, global aspirational goal for advocacy cannot be achieved at the cost of substantial misclassification, with potentially harmful implications, during practical usage.

## Rebuttal (Borghi)

The Committee on the Rights of the Child, through Article 24 of the 1989 Convention on the Rights of the Child, addresses the right of the child to enjoy the highest attainable standard of health and calls for countries to recognize the advantages of breastfeeding [25]. The WHO Child Growth Standards, to date, are the only existing references that are based on the internationally recommended infant and young children feeding practices. It is well known that, for children aged <5 y, adequate feeding is one of the main drivers to promote optimal physiological growth. If those standards cannot consistently predict a biological construct representing the balance between nutritional intake and requirement, which is not even defined for the concerned age group, it seems unlikely that country-specific references will.

It is not difficult to understand the challenges behind designing a study that follows these internationally agreed feeding practices from birth to ≥2 y of age, and the possibility of that happening, especially in countries where inadequate conditions that affect child growth are predominant, is nearly nil. Having country-specific standards will undermine the impact of known contextual factors that affect growth. As an example, Sachdev states that “the selected affluent populations may be more susceptible to overnutrition,” which is clearly a question of the relative measure of “overweight,” where BMI distribution is at lower levels compared with the global standards. If the measure of an indicator does not follow a standard definition, inevitably, comparisons among populations will not be interpretable. Sachdev also cites his recent publication [19] referring to the WHO Growth References for School-age Children and Adolescents [26], covering ages between 5 and 19 y, as “WHO standards.” Without delving into discussions of other age groups beyond the scope of the present paper, it is worth clarifying that there are no standards for those age groups.

Global standards serve as a basis for countries to identify populations exposed to factors adverse to health outcomes and trigger corrective actions. The WHO Child Growth Standards went through many years of scrutinization, with many countries assembling domestic expert committees to consider their pros and cons. To date, >160 countries have endorsed and are using them to assess their children’s growth patterns and define their populations’ nutrition status, which is a testament to their acceptance and validity. Global monitoring is based on those standards to ensure comparability across countries with respect to their nutritional status based on anthropometric indicators.

## Acknowledgments

EB is thankful to Mercedes de Onis for her legacy on evidence building in the field, which supported this piece, and to Francesco Branca, Edward Frongillo, and Richard Kumapley for their feedback on earlier drafts of this manuscript. HSS is grateful to Prof. Anura V. Kurpad, Prof. Tinku Thomas, and Dr. Santu Ghosh for their constructive feedback on earlier drafts of this manuscript.

## Author contribution

EB wrote the main argument and rebuttal; HSS wrote the refutation; all authors read and approved the final manuscript.

## Conflicts of interest

EB works for the WHO, which recommends the global Child Growth Standards for all countries. HSS declares no conflicts of interest.

## Funding

The authors reported no funding received for this study.

### Disclaimer

This article series is designed as an Oxford-style debate. As such, participants are required to argue pro- and con- positions, even when that opinion may differ from their own. The views expressed in this debate do not necessarily reflect the opinion of the participants, *The American Journal of Clinical Nutrition*, or the American Society for Nutrition.
